# Long-term imaging of the photosensitive, reef-building coral *Acropora muricata* using light-sheet illumination

**DOI:** 10.1038/s41598-020-67144-w

**Published:** 2020-06-25

**Authors:** Pierre Philippe Laissue, Loretta Roberson, Yan Gu, Chen Qian, David J. Smith

**Affiliations:** 1 School of Life Sciences, University of Essex, Colchester, CO4 3SQ UK; 2 The Bell Center, Marine Biological Laboratory, 7 MBL Street, Woods Hole, MA 02543 USA; 3 MRC National Institute for Medical Research, Mill Hill, London, NW7 1AA UK; 4 School of Life Sciences, University of Sussex, Brighton, BN1 9QG UK; 5 AstraZeneca, 310 Milton Rd, Milton, Cambridge, CB4 0WG UK

**Keywords:** Light-sheet microscopy, Conservation biology, Sea anemone, Optical imaging

## Abstract

Coral reefs are in alarming decline due to climate emergency, pollution and other man-made disturbances. The numerous ecosystem services derived from coral reefs are underpinned by the growth and physical complexity of reef-forming corals. Our knowledge of their fundamental biology is limited by available technology. We need a better understanding of larval settlement and development, skeletogenesis, interactions with pathogens and symbionts, and how this biology interacts with environmental factors such as light exposure, temperature, and ocean acidification. We here focus on a fast-growing key coloniser, *Acropora muricata (Linnaeus, 1758)*. To enable dynamic imaging of this photosensitive organism at different scales, we developed light-sheet illumination for fluorescence microscopy of small coral colonies. Our approach reveals live polyps in previously unseen detail. An imaging range for *Acropora muricata* with no measurable photodamage is defined based upon polyp expansion, coral tissue reaction, and photobleaching. We quantify polyp retraction as a photosensitive behavioural response and show coral tissue rupture at higher irradiance with blue light. The simple and flexible technique enables non-invasive continuous dynamic imaging of highly photosensitive organisms with sizes between 1 mm^3^ and 5 cm^3^, for eight hours, at high temporal resolution, on a scale from multiple polyps down to cellular resolution. This live imaging tool opens a new window into the dynamics of reef-building corals.

## Introduction

Live imaging is a potent approach for the investigation of fundamental processes and structures of reef-building corals at the microscopic scale. Many microscopy-based studies of corals rely on techniques imaging the calcareous skeleton from which the tissue was removed, or using fixed samples of decalcified coral tissue^1–4^. Therefore, the complex three-dimensional interactions of coral tissue, endosymbiotic algae and aragonite skeleton have been little studied at the tissue and cellular level. Knowledge of such interactions can greatly add to our understanding of fundamental coral biology, which is required to improve strategies for the conservation of coral reefs. In particular, fluorescence microscopy opens up the possibility to use coral tissue autofluorescence as a non-invasive, intrinsic marker of health and disease^5,6^ and the monitoring of chlorophyll autofluorescence of the photosynthetic symbionts embedded in the coral tissue^7,8^. Fluorescent dyes can also be used in live imaging of intrinsic processes such as calcification^9–11^.

**Figure 1:**
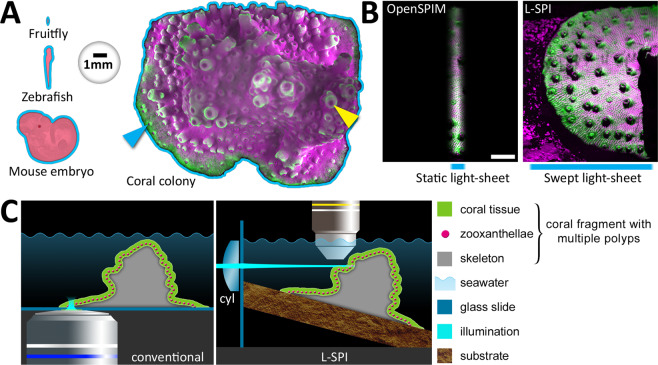
(**A**) Size of commonly used samples and of a small colony of reef-building corals. On the coral colony, the cyan arrowhead indicates the flat, laterally growing edge of the colony, while the yellow arrowhead points to a mature polyp, growing vertically on the upper side of the colony. (**B**) Left side: Width of an OpenSPIM-type static light-sheet. The scale bar is 2 mm. Right side: Our approach (L-SPI) generates a much wider light-sheet, which allows illuminating large parts of a small coral colony in a single FOV. (**C**) Left side: The conventional approach uses inverted microscopy to excite and detect fluorescence in a colony’s growing edge from below. Right side: Our approach (L-SPI) uses light-sheet excitation created by a swept laser beam entering from the left side and passing through a cylindrical lens (‘cyl’), with fluorescence emission detected from above. This enables low-light imaging of complex topology or individual polyps.

The impact of any fluorescence microscopy approach should be as low as reasonably achievable, ideally entirely non-invasive, to allow longitudinal observation and monitoring of reef-building coral colonies at high temporal and spatial resolution. This contributes greatly to the still technically challenging maintenance of healthy reef-building colonies *ex situ* over long periods of time for experimental studies^12–15^. So far, only conventional techniques have been used to image live coral fluorescence on the cellular scale. Confocal laser scanning microscopy (CLSM) of live polyps has been used in *Montipora capitata* at different spatial scales to characterize overall diversity of natural fluorescence and to spatially localize the arrangement of fluorescent pigments^5^. Inverted microscopy, in which the sample is imaged from below, has been used to image several microns deep into the calcifying layers^8,10,11,16^. This approach works well when the main interest lies in the calcification of the flat bottom layer. However, this approach is not well suited for large samples with complex three-dimensional growth such as small coral colonies. Compared to model organisms commonly used in fluorescence microscopy, coral colonies with multiple polyps are considerably larger (Fig. 1A). We chose light-sheet fluorescence microscopy, also called Selective Plane Illumination Microscopy (SPIM,^17^), a versatile technique for samples that cannot be mounted between glass^18^. For large coral samples, we created a very wide light-sheet to capture a large field of view (FOV). The common OpenSPIM-type static Gaussian light-sheet^19^ is around one millimetre wide. It thus reveals only a small part of a coral colony (Fig. 1B, left side, short blue bar). In comparison, by sweeping the beam laterally, our light-sheet can be made as wide as two centimetres (Fig. 1B, right side, wide blue bar). We thus call it the large selective plane illuminator (L-SPI).

The key strength of light-sheet fluorescence microscopy is the ability to minimise photodamage during live imaging. This was an essential consideration for observing the photosensitive species *Acropora muricata*. In conventional microscopy, fluorescence in tissue, cells or aragonite skeleton is excited and detected through the same (inverted) objective (Fig. 1C, ‘conventional’). The consequence is that for each focal plane, the entire sample is illuminated. This makes conventional fluorescence microscopy techniques not well suited for long-term imaging of photosensitive samples. By contrast, the excitation pathway in light-sheet microscopy is uncoupled and comes in from the side through a cylindrical lens (Fig. 1C, ‘L-SPI’). It illuminates only the focal plane. This enables live imaging in three spatial dimensions at minimal light exposure.

We used small colonies of *Acropora muricata*^20^ as an experimental model for coral development and behaviour. Corals of the genus *Acropora* are fast-growing, ecologically crucial key architects of coral reef ecosystems, greatly contributing to their complex three-dimensional structure^21^. They can also recover rapidly from environmental disturbances, making them important re-colonisers^22^. Rather than using cell or tissue culture-based samples^23^, single primary polyps^10,11^ or single polyps obtained through bail-out techniques^8^, we used small coral colonies with multiple polyps and a shared gastrovascular cavity. They allow the study of systemic responses in an unstressed, fully established colony. Our non-invasive observation technique for fluorescence expands the live-imaging toolbox to photosensitive species and long-term observation of chlorophyll fluorescence in algal symbionts.

Using light-sheet microscopy provides a further advantage: It is ideally suited for darkfield observation^24–26^. Darkfield microscopy forms an image by detecting scattered light that is refracted, diffracted or reflected off the sample. Since the illuminating light is already perpendicular to the optical axis of detection, light-sheet microscopy inherently provides darkfield illumination. By contrast, conventional single-objective approaches require additional methods to enable darkfield illumination (by blocking out the central light rays along the optical axis of the microscope). Darkfield microscopy can detect submicroscopic structures such as nanoparticles^27^ and has been used in coral research to visualise bacterial shedding^28^ and flow fields caused by ciliary beating^29^. We used darkfield illumination in the L-SPI to acquire high-contrast images of unstained, non-fluorescent structures, such as the aragonite skeleton. To determine if this technique could be used in species with little or no tissue autofluorescence, we imaged colonies of the temperate coral *Astrangia poculata*. This ability to image non-fluorescent structures and species at low irradiance makes darkfield microscopy a highly useful method to complement non-invasive fluorescence microscopy using the same light-sheet approach.

## Results

### Morphology of large *Acropora muricata* colonies at multiple scales

**Figure 2:**
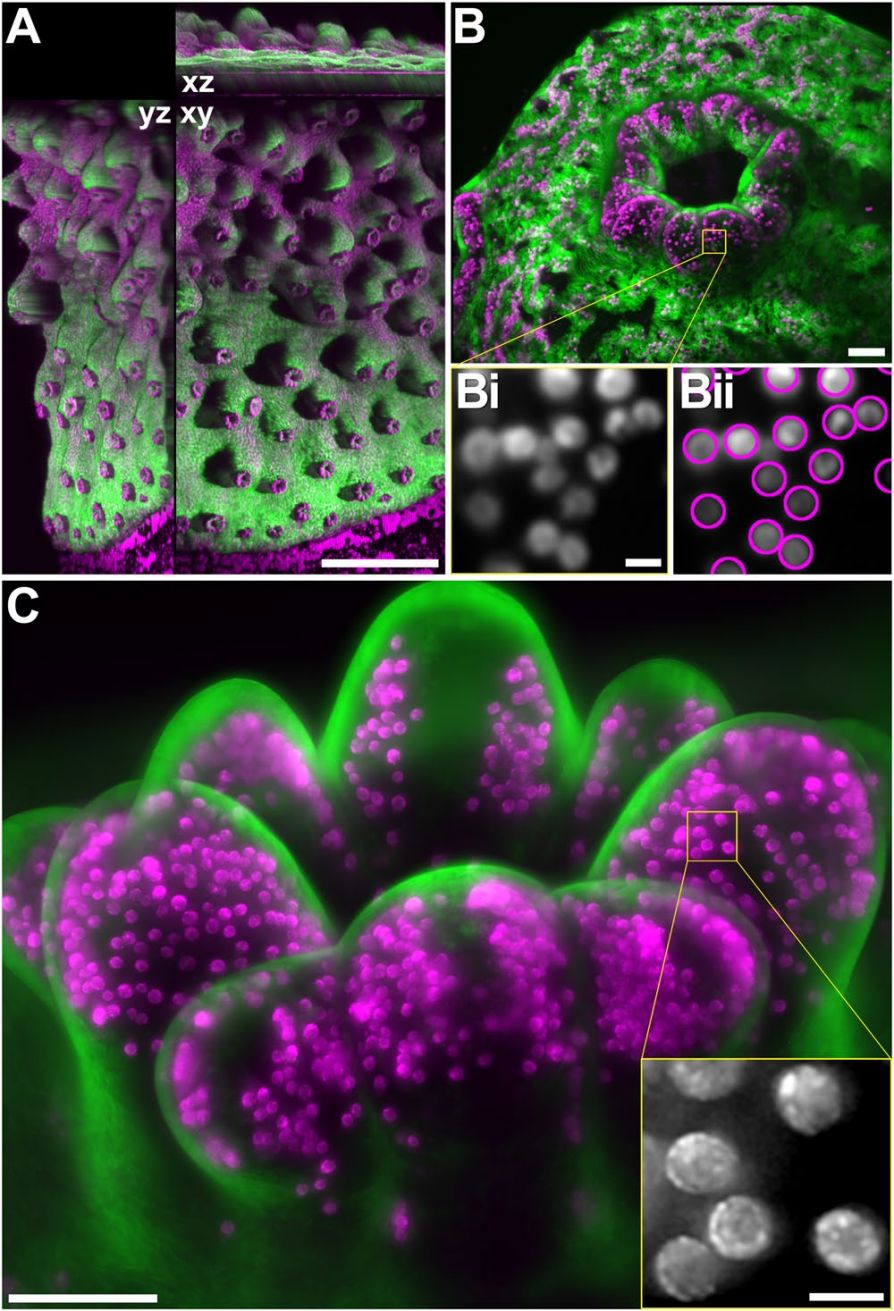
Volumes of photosensitive *Acropora muricata* obtained with the L-SPI. In all images, coral tissue autofluorescence is green, while magenta shows the chlorophyll autofluorescence of the symbiotic zooxanthellae. (**A**) Large field of view (FOV) of multiple polyps in a coral colony. The orthogonal sections (xy, xz and yz) show the topology of its growth. Scalebar 5 mm. (**B**) Larger magnification (10X) of a coral polyp. Scalebar 100 µm. Bi) Close-up of a small region (yellow box in (**B**) showing individual zooxanthellae. Scalebar 10 µm. Bii) Automated identification of individual zooxanthellae. (**C**) A small, developing coral polyp (imaged at 20X) showing the distribution of symbiotic algae embedded in the tentacles. Scalebar 100 µm. Inset: High magnification view of zooxanthellae showing subcellular detail. Scalebar 10 µm.

Width and waist (the waist being the thinnest part of a light-sheet along its direction of propagation) of the L-SPI light-sheets were adapted to cover samples of different sizes. This enabled imaging of a coral colony at multiple scales (Fig. 2). Wide light-sheets (2 cm width) with a large waist (20.7 ± 0.8 µm standard deviation (SD)) were used to provide an overview of large parts of a colony in a single field-of-view (FOV). Figure 2A shows multiple polyps in a FOV of 26 mm × 19 mm. These datasets are acquired in three spatial dimensions (x, y and z), also called z-stacks. They reveal the topology of the cup-shaped corallites. A corallite is the protective, skeletal cover into which a single polyp can retract^1,30,31^. Corallites are very flat at the growing edge, and rise up with increased distance from the edge (which correlates with their advanced developmental stage). Since the coral skeleton is entirely opaque, a certain amount of shadowing cannot be avoided. Note that all polyps have emerged. The L-SPI scanning process is shown in Supplementary Video S1. All Supplementary Videos are listed in Supplementary Table 1. The different lateral resolutions and light-sheet dimensions are summarised in Supplementary Table 2.

Using a narrower light-sheet with thinner waist (11.7 ± 0.8 µm SD) allows focussing on a single coral polyp and large parts of its surrounding tissue (Fig. 2B). The coral tissue has strong green autofluorescence and a fibrous structure. It clings to the side of the prominent skeletal spines^1,30,31^. Zooxanthellae are arranged in wavy bands within the tissue. At this magnification, single zooxanthellae in the polyp tentacles are resolved (Fig. 2Bi). This enables automated counting using simple image processing (Fig. 2Bii). A small polyp in an earlier stage of development is shown at higher resolution in Fig. 2C (Supplementary Video S2). Subcellular structures of zooxanthellae are visible at this scale. Zooxanthellae have a diameter of 8.2 ± 0.1 µm (n = 100; 95% confidence interval (CI) 7.98–8.39). They are densely packed in the budding tentacles of the developing polyp, arranged as a single-cell layer on each side of a tentacle (as seen in Fig. 2C and the corresponding Supplementary Video S2). On average, such a developing tentacle had 57 ± 3 zooxanthellae inside it (n = 15). In mature polyps, tentacles are more elongated and pointy, and the zooxanthellar layers often join up to form a funnel-shaped single-cell layer of zooxanthellae (as seen in Supplementary Video S6). Such a mature tentacle had on average 112 ± 9 zooxanthellae inside it (n = 14).

### The rate of polyp expansion depends on excitation light irradiance in *Acropora muricata*

**Figure 3:**
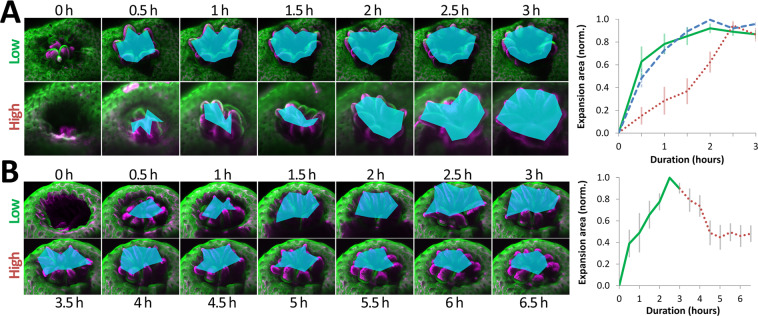
Polyp emergence and retraction under low and high irradiance. (**A**) Images of two polyps in low and high irradiance conditions. Emergence is quantified using the area determined by connecting the tips of tentacles. At low irradiance (18.4 mW/cm^2^), polyps have nearly fully emerged after one hour. At high irradiance (82.6 mW/cm^2^), polyp emergence takes more than twice as long. This is quantified in the line graph on the right. Green continuous line: Polyp emergence at low irradiance. Red dotted line: Polyp emergence at high irradiance. Blue dotted line: Polyp emergence in low, white light. Error bars show the standard error of the mean (SEM). (**B**) Images of a polyp switching from low to high irradiance. The polyp emerges at low irradiance (9.2 mW/cm^2^) and retracts in high light (59.7 mW/cm^2^). This is quantified in the line graph on the right. Green continuous line: Polyp emergence at low irradiance. Red dotted line: Polyp contraction at high irradiance. Error bars show the SEM.

We have previously suggested that the expansion and contraction of polyps can be used to determine the ranges for non-invasive and damaging levels of excitation light used for microscopic observation of *Acropora muricata*^32^, and quantify the ranges in this study. Polyps were retracted at the start of an imaging experiment due to transfer of the coral fragment from the main aquarium to the observation vessel. As shown in Fig. 3, connecting the tips of a polyp’s tentacles was used to determine the area of expansion. Seven polyps in three replicates were analysed for each condition. At an irradiance of 18.4 mW/cm^2^, polyps had expanded to a median value of 93% within 90 minutes (95% CI 0.78–1.00; Fig. 3A top row, green continuous line in graph; Supplementary Video S3). This was nearly identical to polyps emerging in low white light (using brightfield microscopy), which had expanded to 92% in the same time (95% CI 0.79–1.00, p = 0.776; blue dashed line in graph; Supplementary Video S4). By contrast, at 82.6 mW/cm^2^ irradiance, expansion within the same time was only 41% (95% CI 0.14–0.64, p = 0.008; Fig. 3A, bottom row; red dotted line in graph; Supplementary Video S5). Photobleaching of the coral tissue was also far more pronounced at high irradiance, losing 64% ± 3% of its original intensity after three hours, compared to a mere reduction of 9% ± 3% at low irradiance (Supplementary Fig. S1).

We further lowered the high and low irradiance values (59.7 mW/cm^2^ and 9.2 mW/cm^2^, respectively) to identify the lower end of each range. 9.2 mW/cm^2^ produced sufficient image contrast. As the aim of this study was to identify the lowest possible irradiance conditions for long-term, volumetric, continuous fluorescence microscopy of *Acropora muricata*, we continued to use this more conservative value for non-invasive imaging. Polyps which had expanded at this low end of excitation irradiance (9.2 mW/cm^2^) would retract at high irradiance (59.7 mW/cm^2^) (Fig. 3B; Supplementary Video S6). To check if the corals were just reacting to changes in irradiance, we switched from high (59.7 mW/cm^2^) to low irradiance (9.2 mW/cm^2^) after three hours. However, this did not cause polyps to contract (Supplementary Fig. S2).

The above ranges for low and high irradiance (9.2 to 18.4 mW/cm^2^) and (59.7 to 82.6 mW/cm^2^) provided an estimate of tolerable versus excessive levels of blue light for fluorescence excitation. The different irradiance conditions used in this study are summarised in Supplementary Table 3. The corresponding powers and biological replicates are listed in Supplementary Table 4.

### Non-invasive continuous imaging for at least six hours does not cause light-induced stress

**Figure 4:**
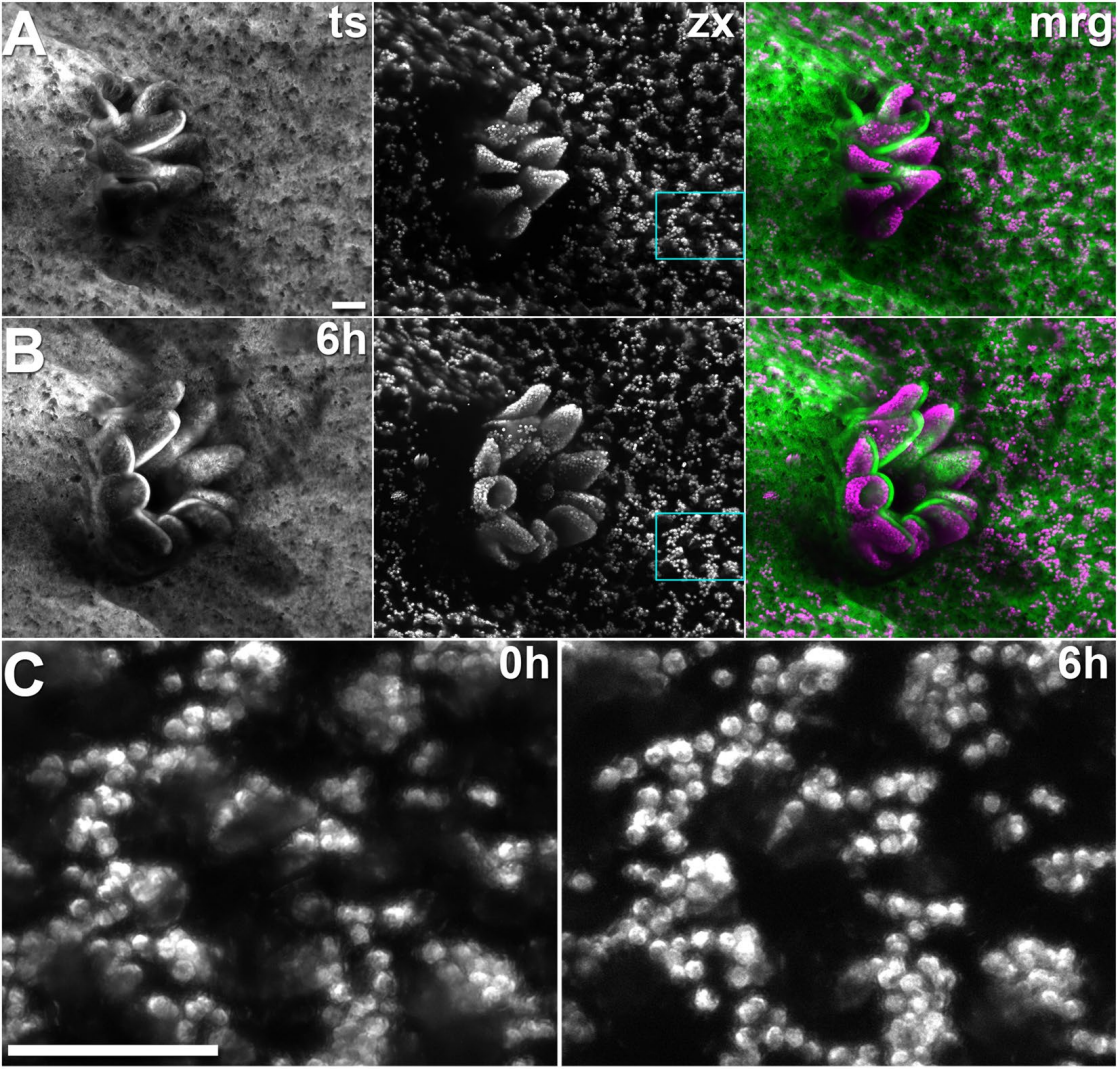
Still frames from sequential z-stacks recorded over six hours at low light (9.2 mW/cm^2^). (**A**) Polyp at start of acquisition. From left to right: Coral tissue autofluorescence (ts), chlorophyll autofluorescence of the zooxanthellae (zx), and merged image (tissue green, zooxanthellae magenta). (**B**) The same polyp after six hours of continuous image acquisition. (**C**) Close-up of the cyan boxes in A and B showing the unchanged arrangement of the zooxanthellae inside the tissue. Scalebars 100 µm.

The conservative irradiance value which allowed rapid polyp expansion (9.2 mW/cm^2^) was used to extend exposure times. Long time-lapse recordings of sequential z-stacks of coral tissue and zooxanthellar chlorophyll autofluorescence were taken. Figure 4 (Supplementary Video S7) shows the typical dynamics of a coral polyp of *Acropora muricata* over six hours of continuous imaging at low irradiance. The polyp expanded rapidly within half an hour, and stayed expanded for the rest of the time-lapse acquisition. Such unchanged polyp expansion was measured in three replicates over 6.5 hours (Supplementary Fig. S3).

Distribution of zooxanthellae in the field of view changed very little over the duration of the time-lapse: The same arrangement of zooxanthellae in the field of view can be identified after six hours of continuous image acquisition (Fig. 4C, Supplementary Video S8). Individual zooxanthellae were tracked to show the minimal displacement (Supplementary Video S9). This tracking analysis was performed in three replicates over 8.0 ± 1.5 hours continuous image acquisition; the median value for the displacement of zooxanthellae (n = 3882) was 1.95 µm, with a median absolute deviation of 1.09 µm (95% CI 1.87–2.01).

Since fluorescence intensity and photobleaching correlate with the generation of reactive oxygen species, photobleaching is a useful proxy for the semiquantitative assessment of phototoxicity^32–34^. In low irradiance conditions, photobleaching of the coral tissue was minimal (10% reduction in fluorescence intensity) and reached a plateau after one hour (see also Fig. 7C). In addition, daily visual inspection of the coral fragments after imaging showed no signs of delayed photodamage, such as paling of the brown, zooxanthellae-studded tissue, tissue rupture or tissue loss. The growth rate of non-invasively imaged colonies was determined over 20 ± 1.5 days and compared to controls imaged with low-light brightfield microscopy (Supplementary Fig. S4). Imaged samples had an average growth rate of 75.4 ± 6.8 µm/day, non-imaged samples 76.5 ± 9.0 µm/day. We hypothesised that these various characteristics signified the absence of light-induced stress. A median value of eight hours (mean value 8.3 ± 0.8 h) continuous image acquisition was achieved (n = 6) using 9.2 mW/cm^2^ irradiance. The average exposure was 184 ± 20 mJ. Samples imaged within this non-invasive range were re-used for imaging at least once. Between imaging sessions, a fragment was kept in the aquarium for at least one day. Given a typical polyp size of 1 mm × 1 mm × 0.8 mm, this non-invasive irradiance results in a very low energy density of 0.23 nJ/μm^3^. With an average of 220 volumes over an 8 h period of observation, this implies an exposure of 836 µJ per stack and an energy density of 1.0 pJ/µm^3^ per stack.

**Figure 5:**
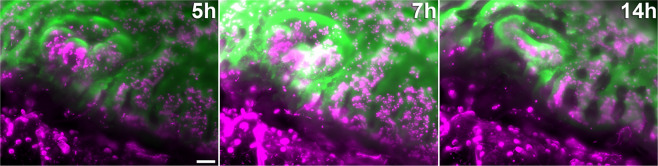
Still frames from a 16 hour continuous recording in excessive light conditions (47.8 mW/cm^2^). Coral tissue autofluorescence (green) and chlorophyll autofluorescence (magenta) are shown after five hours of continuous imaging (left). At seven hours (middle), a surge in autofluorescence intensity was visible. This was followed by tissue rupture (occurring between nine and eleven hours), which led to the growing edge dissolving after 14 h (right). Scale bar 100 µm.

**Figure 6:**
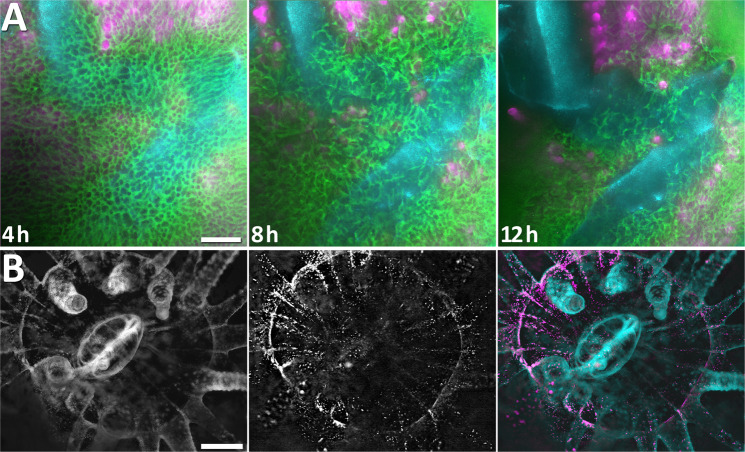
(**A**) Still frames from a 12 hour continuous recording in excessive light conditions (47.8 mW/cm^2^). Coral tissue autofluorescence (green) and chlorophyll autofluorescence (magenta) are shown after four hours of continuous imaging (left). The two cyan areas indicate the non-fluorescent coral skeleton underneath the tissue, visualised using darkfield imaging. At eight hours (middle), tissue rupture occurs. The ordered structure of the coral tissue autofluorescence is disrupted, and the underlying skeleton (cyan) revealed. Rupture continued to 12 hours until much of the underlying skeleton is visible. Scale bar 100 µm. (**B**) Darkfield imaging is used to reveal the non-autofluorescent coral tissue (left) in the Northern Star coral (*Astrangia poculata*), a species with no intrinsic fluorescence. Zooxanthellae are shown in the middle image. Non-fluorescent tissue (cyan) and zooxanthellae (magenta) are combined in the image on the right. Scale bar 500 µm.

**Figure 7:**
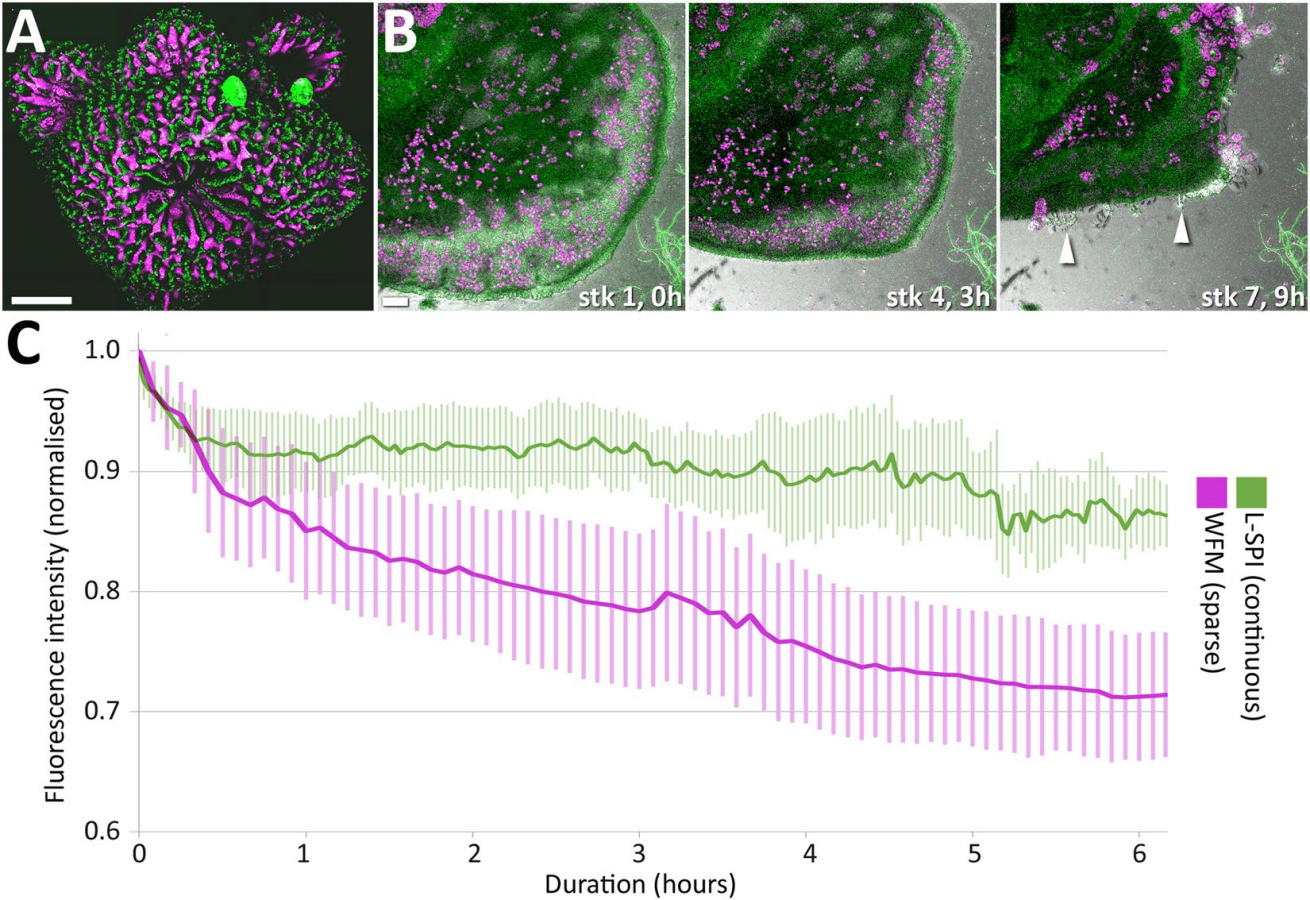
(**A**) Polyps at the tip of a coral branch imaged with confocal laser scanning microscopy (CLSM). The large terminal polyp in the image centre, and the three smaller polyps in the upper half of the image, had all fully retracted into their corallites. Scalebar 500 µm. (**B**) Severe retraction and rupture of coral tissue at the growing edge in A. muricata. At timepoint 0 in the first z-stack (stk 1, 0 h), the tissue of the growing edge was expanded. Three z-stacks and three hours later (stk 4, 3 h), the edge had retracted. Scans were then further reduced to one scan every two hours. However, after seven stacks (stk 7, 9 h), the tissue started to rupture, exposing skeletal elements (white arrowheads) and compressing zooxanthellae in the gastrovascular cavity. Scalebar 100 µm. (**C**) Comparison of photobleaching using sparsely sampled widefield fluorescence microscopy (WFM, magenta) and L-SPI (green) over six hours. The graph shows the average of three replicates for each condition. Error bars indicate SEM. Despite low irradiance (1.0 mW/cm^2^) and reduced volumetric and temporal imaging for WFM, photobleaching was 15 ± 3% higher after six hours compared to the L-SPI.

### High irradiance leads to tissue rupture

Photodamage was far more severe when high excitation irradiance was used from the start. Figure 5 (Supplementary Video S10) shows that this increased irradiance eventually caused tissue rupture. Using 47.8 mW/cm^2^ irradiance for continuous image acquisition, no adverse reactions were observed for the first five hours, apart from pronounced photobleaching of the coral tissue. After seven hours, there was a surge in coral tissue and chlorophyll autofluorescence, followed by tissue contraction and rupture starting at nine hours and continuing through to 14 hours. Three replicates were imaged at high irradiance (47.8 mW/cm^2^) for an average of 8.7 ± 0.3 hours. In all cases, the illuminated area was irreversibly damaged and the entire colony perished within two days. This started with tissue rupture in the field of view (Figs. 5 and 6, Supplementary Video S10) and subsequent tissue loss, which progressed from the injured (imaged) location on the sample across the rest of the fragment over the course of one to two days until the small coral fragment was devoid of living coral tissue and consisted solely of aragonite skeleton.

### Darkfield imaging reveals non-fluorescent structures

Darkfield imaging revealed non-fluorescent coral skeleton in acroporid samples exposed to high irradiance, showing how the tissue stretches and ruptures over skeletal parts (Fig. 6A). This approach can further be used for coral species that do not possess tissue autofluorescence, such as the northern star coral *Astrangia poculata*. We thus used darkfield imaging to visualise its tentacles and mouth (Fig. 6B), which is highly useful to put the chlorophyll autofluorescence of its endosymbiotic algae in context. This also opens up the possibility of adding fluorescent dyes to this kind of sample, revealing staining properties that would otherwise be masked by tissue autofluorescence.

### Conventional fluorescence microscopy methods cause more photobleaching and photodamage compared to the L-SPI

Using confocal laser scanning microscopy (CLSM) for image acquisition of *Acropora muricata* in three spatial dimensions invariably caused polyps to retract deeply into their corallites, even at a very low excitation power of 1.1 µW (Fig. 7A, Supplementary Video S11). Observing polyp dynamics in *Acropora muricata* was not possible using CLSM. Attempting longer-term time-lapse recordings at the flat, laterally growing edge of the colony (for reference, see Fig. 1A) resulted in marked tissue contraction and eventual rupture (Fig. 7B). This is not surprising since CLSM produces a focal laser point with very high irradiance: Using a 10x lens with a numerical aperture (NA) of 0.3 and very low laser excitation (1.1 µW) results in 34′414 mW/cm^2^, which is larger by a factor of 3′741 compared to the light-sheet’s irradiance at the non-invasive level (9.2 mW/cm^2^). In conclusion, in the case of *Acropora muricata*, CLSM provided live morphology in three spatial dimensions (3D) due to its optical sectioning capability, but the inflicted photodamage limited acquisition to a single timepoint. All samples (n = 6) imaged by CLSM perished within one to two days after imaging, in the same way as described above for high-irradiance light-sheet samples.

We then compared the L-SPI to widefield epifluorescence imaging (WFM). We wanted to see if WFM might be able to perform as well as light-sheet microscopy purely in terms of photobleaching. To reduce light exposure as much as possible for WFM, we used lower magnification (CFI Plan Apochromat λ 4X, NA 0.2; Nikon Corporation, Tokyo, Japan), enabling large 50 µm z-steps, so that a polyp was covered using only 11 to 13 z-planes. We further limited acquisition of these z-stacks to 5 minutes intervals, resulting in longer dark phases to further minimise photobleaching. At 1.0 mW/cm^2^, irradiance was ten times lower compared to LSFM. However, despite these efforts to minimise exposure in WFM, photobleaching of the coral tissue was higher compared to the L-SPI (Fig. 7B, Supplementary Video S12). The L-SPI covered six times more z-planes, nearly two and a half times higher time resolution, and at least one and a half times higher lateral resolution. Thus, the L-SPI, compared to sparse WFM, provides over 22 times more information while still causing 15% ± 3% less photobleaching after six hours (Fig. 7C; 86% and 71% of initial fluorescence intensity, respectively; n = 3 for each condition).

## Discussion

We have developed and validated a novel light-sheet approach for non-invasive fluorescence and darkfield microscopy of reef-building corals. The instrument is capable of long-term three-dimensional imaging in photosensitive *Acropora muricata* without any measurable adverse reactions. This allowed us to observe the dynamics of small coral colonies in unprecedented temporal, spatial and spectral detail. We depict zooxanthellae arranged in bands within the coral tissue, and as a dense layer in polyp tentacles. We demonstrate that polyp retraction is a photosensitive response, and define a non-invasive range for fluorescence excitation. A median value of 8 hours (mean value 8.3 ± 0.8 h) continuous image acquisition was possible using 488 nm excitation at 9.2 mW/cm^2^ irradiance. This resulted in an average exposure of 184 ± 20 mJ, with coral colonies showing no signs of short-term light-induced stress or long-term photodamage. Substantially exceeding the non-invasive level led to contraction and rupture of the coral tissue.

Polyp expansion and retraction is a fundamental behaviour in cnidarians^37^. During daytime, branched corals with small polyps expand their tentacles to expose photosynthetic symbionts, localized within the tentacles, to light^38–40^. Conversely, they retract their tentacles in high light conditions to protect the photosynthetic symbionts from damaging irradiance^41–44^. Maximum photobehaviour response has been demonstrated in the blue/green zone^38,45^. Our observations on photosensitive polyp dynamics are consistent with these published findings. Levy and coworkers^46^ report average values at 5 m depth in Gulf of Eilat of around 12 mW/cm^2^ from about 11 am to 4 pm. Our non-invasive imaging range (9.2 mW/cm^2^ (equalling 375 µmol/m^2^/s photosynthetic photon flux density) for an average of 8.3 ± 0.8 hours) broadly agrees with this value. It should also be noted that the 9.2 mW/cm^2^ are peak values, not average values, for the region illuminated (between 80 ms and 1 s per image) by the light-sheet. The light-sheet passing over the coral is not dissimilar to the effect of transient peak values of sunlight from water lensing, caused by ripples and waves at the top of the water.

Upon settling at the beginning of an imaging session, coral tissue often appears to swell within the first ten to twenty minutes. This is likely caused by an influx of seawater into the gastrovascular cavity. This tissue inflation also appears to contribute to a drop in autofluorescence intensity. Thus, the drop in coral tissue autofluorescence observed at low irradiance levels using the light-sheet may be only partly due to actual photobleaching.

Combining light-sheet microscopy with non-fluorescent modalities such as optical projection tomography is a highly useful approach.^47,48^ We here used darkfield imaging to complement light-sheet fluorescence. In staghorn corals, this enabled the visualisation of non-fluorescent coral skeleton beneath the autofluorescent coral tissue. Darkfield microscopy also opens up the possibility to use non-fluorescent coral species. For the northern star species *Astrangia poculata* with no coral tissue autofluorescence, darkfield microscopy circumvented the need to use a fluorescent dye to visualise its tentacles and mouth. This allowed clear localisation of the chlorophyll autofluorescence of its endosymbiotic algae.

The L-SPI performed better than the conventional fluorescence microscopy methods WFM and CLSM. Despite using low excitation power, *Acropora muricata* was particularly susceptible to CLSM. Quantitative comparisons to previous studies using conventional microscopy (WFM, CLSM and spinning disk confocal) for live imaging of *Montipora capitata*^5,35^, *Acropora digitifera*^10^, *Pocillopora damicornis* and *Stylophora pistillata*^7–9,11,16,36^ were not possible as no power measurements were reported. Substantial differences in phototolerance may exist between different species.

The instrumental setup for the L-SPI is simple and flexible. It performs best with highly photosensitive organisms, and can image small to large sample volumes (1 mm^3^ to 5 cm^3^). These attributes enable studying the responses of tissue and symbionts on different spatial scales, at high temporal resolution and over several hours. This live imaging tool will continue to provide a new window into the dynamics of reef-building corals.

## Materials and Methods

### Aquarium husbandry

Colonies of *Acropora muricata* (Linnaeus, 1758) were obtained from cultured stock (Tropical Marine Centre, Chorleywood, UK) and kept within a closed aquarium system circulating 1700L of artificial salt water. Salt water was prepared using reverse osmosis water mixed with a commercial synthetic sea salt (H2Ocean Pro+ Reef Salt, Charterhouse aquatics, UK). Water parameters were maintained at the following levels (± SD): salinity 35 ± 0.5 ppt, temperature 26.8 ± 0.5 °C, pH 8.2 ± 0.3, ammonium <0.01 mg/L, nitrite <0.01 mg/L, phosphate <0.25 mg/L, nitrate <10 mg/L, calcium 420 ± 40 ppm, alkalinity 2.7 ± 0.5 mEq. Temperature and salinity were tested daily using a digital hand held thermometer (tolerance ± 0.2 °C, E.T.I Ltd Reference thermometer) and refractometer. All other parameters were tested once per week using API colour change test kits (NT Laboratories Ltd., Chorleywood, UK). Weekly water changes were performed to maintain parameters and additional sodium carbonate, sodium hydrogen carbonate and calcium chloride were added to sustain alkalinity and calcium levels. Photosynthetic photon flux density was measured using a biospherical PAR sensor (LI-250A, LI-COR Biosciences, Lincoln, Nebraska, USA) and maintained at 280 ± 50 µmol/m^2^/s using four 54 W metal halide lamps for actinic light (Growth Technology Ltd., Somerset, UK). The light regime was on a 12:12 light:dark cycle. *Astrangia poculata* colonies were collected in Woods Hole, MA (Scientific Permit Number 152087) at a depth of 10 m, transported to the Marine Biological Laboratory, and placed in running seawater. Water temperature was maintained at 18 °C and salinity was 32 ppt during the experiment. Daytime light levels were between 10–15 µmol/m^2^/s. All corals were fed newly hatched *Artemia* three times per week. Fluorescence microscopy experiments on coral fragments were performed in the daytime, during the light cycle. For brightfield microscopy, a tungsten lamp at 234 ± 23 µmol/m^2^/s was used. Fluorescence microscopy experiments (both L-SPI and conventional) on coral fragments were performed in the daytime, during the light cycle.

### Sample preparation

One large coral colony of *Acropora muricata* was fragmented into 26 smaller colonies (with an average of 30 ± 1 mm length, 23 ± 1 mm width, 15 ± 2 mm height), each with multiple polyps (259 ± 53). These fragments were glued to various substrates pre-treated with crustose coralline algae using veterinary-grade cyano-acrylate (superglue)^49–51^. Imaging chambers were assembled from large optical-grade microscope slides (Agar Scientific, Elektron Technology Ltd, Essex, UK) using fungicide- and solvent-free silicon (Everbuild Building Products Ltd., Leeds, UK). The small aquaria were perfused at 27 °C using a peristaltic pump. *Astrangia poculata* were kept at room temperature without perfusion.

### Large selective plane illumination

**Figure 8:**
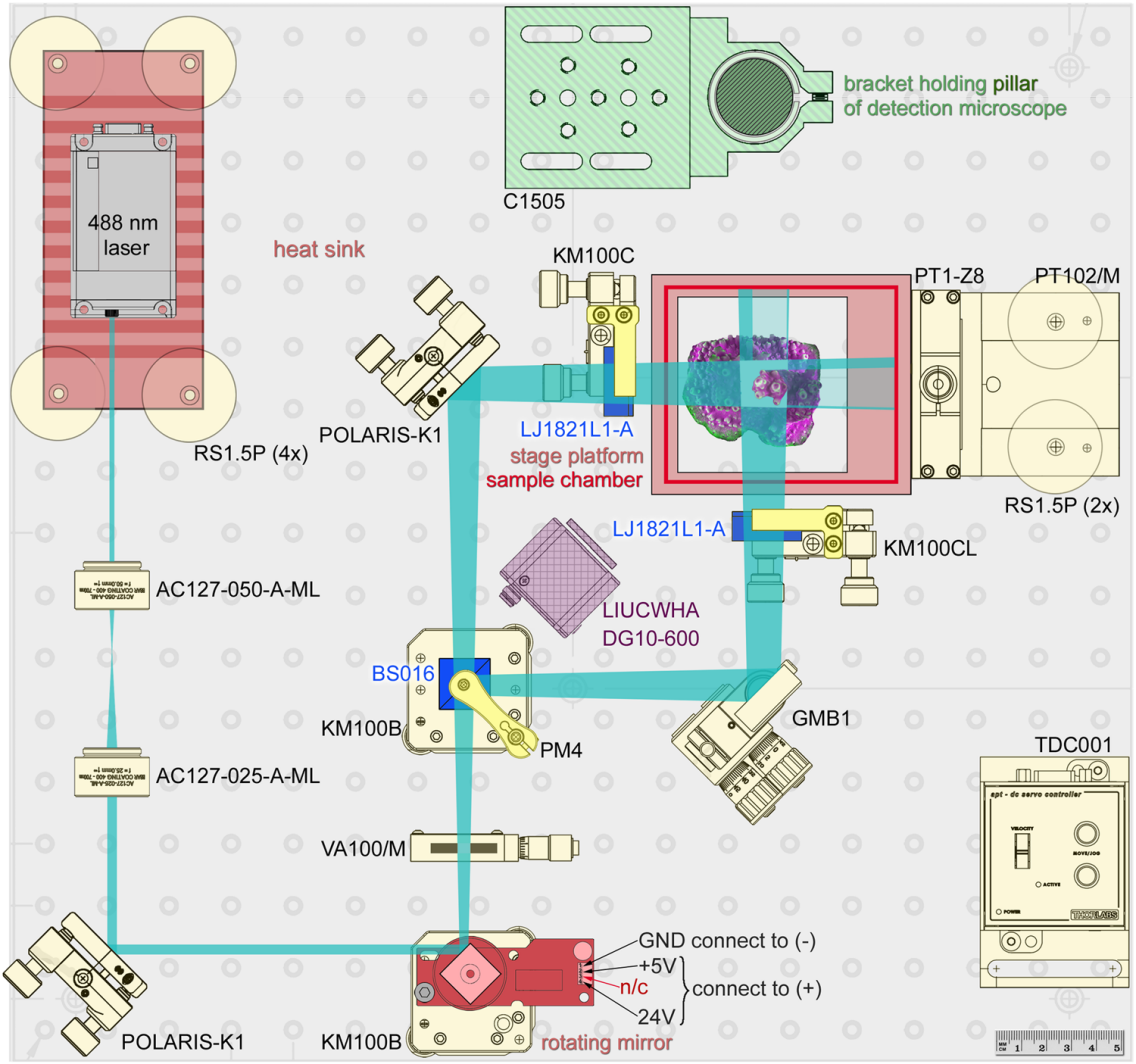
Schematic view of the illumination path for generating two broad light-sheets, as viewed from above. Briefly, the laser beam (top left) is expanded twice and projected onto the rotating mirror, which fans out the beam into a wide light-sheet. The light-sheet passes through a 50/50 non-polarising beamsplitter, generating two light-sheets of identical dimensions. The coral sample is then stepped through these light-sheets while images are acquired from above using any upright micro- or macroscope. The components list is provided in Supplementary Table 5.

The L-SPI setup for coral *in vivo* imaging is detailed below (Fig. 8). Components and requisite weblinks are listed in Supplementary Table 5. The instrument was originally based on the OpenSPIM design^19^. A 488 nm Optically Pumped Semiconductor Laser (OBIS 488 nm LS 100 mW, Coherent, Inc., Santa Clara, CA, USA) was controlled using an Arduino microcomputer and the open source software µManager^52,53^. The beam was expanded twice and reflected onto a rotating mirror (AN8248NSB, Panasonic Corporation, Osaka, Japan) extracted from an old laser printer (for an equivalent rotating mirror, see Supplementary Table 5). The robust brushless motor was powered by a 375 W linear DC variable voltage power supply (Maplin Electronics, Wombwell, UK). The rotating mirror fans the beam out laterally, creating a large light-sheet. Its width was controlled using an adjustable mechanical slit. A 50/50 beamsplitter combined with a prism split the light sheet in opposite directions. After each arm passed through a cylindrical lens, the two light sheets were recombined at a right angle, to reduce shadowing without the need to rotate the sample. Small coral colonies were individually stepped through the light-sheets using a motorized translation stage (Thorlabs Inc., Newton, New Jersey, USA).

Blueprint, components and weblinks required to build the instrument presented are provided in this study. The L-SPI has also been developed into a commercial version (Cairn Research Ltd., UK) to allow researchers who do not have the time or expertise to build the instrument described to benefit from its advantages.

### Detection

Images are taken from above using any upright micro- or macroscope. We have used two main approaches: A compound microscope with low (2X–5X) to medium (10X–40X) magnification objectives, and a macroscope (or stereomicroscope) with low magnification objectives (1X–2X) and zoom function. Images with large fields of view were taken using an SMZ25 stereomicroscope (Nikon Corporation, Tokyo, Japan), equipped with P2-SHR Plan Apochromatic 1×(NA 0.156, WD 60 mm) and 2×(NA 0.312, WD 20 mm) objectives. A Retiga 6000 CCD camera (Photometrics, AZ, USA) was used for detection, combined with an OptoSpin filter wheel (Cairn Research, Faversham, UK). Motorised z-stage and cameras were controlled using the open-source software µManager^52^. Higher-resolution images of single polyps were taken using a BX41 compound microscope with water immersion objectives. A table of the different objectives used is provided in Supplementary Table 2.

### CLSM and WFM

A Nikon A1si CLSM (Nikon Corporation, Tokyo, Japan) with a spectral detector unit was used to define the autofluorescence signatures of coral tissue and zooxanthellae. Using an Argon-Ion laser at 488 nm (40 mW, Melles Griot plc, Carlsbad, CA, USA), coral tissue autofluorescence had its emission maximum at 500 nm and was collected using a 525/50 nm filter, while zooxanthellae showed characteristic chlorophyll *a* emission at a maximum of 685 nm, acquired by using a 700/75 nm emission filter. Objectives were CFI Plan Apochromat λ 4X(NA 0.2, WD 20 mm) and CFI PlanFluor 10×(NA 0.3, WD 16 mm). Images were acquired in two channels using one-way sequential line scans. NIS-Elements (version 3.21.03, build 705 LO) was used to acquire images.

WFM was performed using a Nikon Eclipse Ti-E main body and NIS-Elements (version 3.21.03, build 705 LO) for image acquisition. Objectives were a CFI Plan Apochromat λ 4X(NA 0.2, WD 20 mm) and a CFI PlanFluor 10×(NA 0.3, WD 16 mm). Excitation wavelength was 470 ± 10 nm using a pE-2 LED illuminator (CoolLED Ltd., Andover, UK). Hard-coated interference filters (Semrock Inc., IDEX corp., IL, USA) were used. For chlorophyll autofluorescence, these were a chromatic reflector at 665 nm and a long-pass emission filter at 664 nm. For tissue autofluorescence, we used a chromatic reflector at 505 nm and a single band emission filter at 535/40 (Chroma Technology Corp., VT, USA). Images were acquired with a Retiga 6000 CCD camera (Photometrics, AZ, USA).

### Irradiance and power measurements

Irradiance conditions are listed in Supplementary Table 3. Power measurements for all systems were done with an ML9002A optical handy power meter (Anritsu Corp., Japan) measured at the sample. The powers are summarised in Supplementary Table 4.

### Image processing

Image processing was done using the open-source software FIJI^54^. As the L-SPI takes images from a single viewpoint, no fusion of multiple views was required. We used maximum intensity projection (‘Z Project’) to collapse a three-dimensional z-stack into a single image. Different channels were combined in pseudocolours using the ‘Merge channel’ command in FIJI. All datasets were linearly adjusted for contrast and brightness. For zooxanthellae counts in tentacles, we used code published by Obara and coworkers^55^. To track single zooxanthellae and calculate their displacement, the FIJI plugin Trackmate was used^56^. Images were laid out and labelled for publication using Adobe Photoshop CS6 Extended, version 13.0.1 (Adobe Systems Inc, San Francisco, CA, USA). Videos for Supplementary Material were produced in FIJI as uncompressed AVI, then compressed to mp4 format using the open-source video transcoder HandBrake (https://github.com/HandBrake). The workstation was a Dell Precision T7910 XL with an Intel Xeon Processor E5-2620 v3 (6 C, 2.4 GHz, 15 M, 85 W) and an AMD FirePro W7100 8GB graphics card and 32 GB random access memory (RAM).

Polyp emergence and retraction were quantified in the following way: The starting point for assessing a photosensitive response in polyps was the visibility of tentacles emerging from the corallite. Expansion was quantified by connecting the tips of a polyp’s tentacles, providing an area of expansion. A normalised value of one meant full expansion (or highest expansion for the duration of observation), and zero denoted full contraction (or lowest expansion for the duration of observation). A four-fold increase in area was the minimum value to be counted as expansion.

### Statistical analysis

A minimum of three replicates of *Acropora muricata* were used for each experimental condition. The detailed use of replicates is listed in Supplementary Table 4. Reported errors are standard error of the mean (SEM) unless standard deviation (SD) is indicated. For statistical analysis, we used the PlotsOfDifferences web application^57^. P-values were calculated using a randomisation approach^58,59^. Graphs were produced using Excel (Microsoft Corporation, Redmond, Washington, USA).

## Supplementary information

Supplementary information.

Supplementary information1.

Supplementary information2.

Supplementary information3.

Supplementary information4.

Supplementary information5.

Supplementary information6.

Supplementary information7.

Supplementary information8.

Supplementary information9.

Supplementary information10.

Supplementary information11.

Supplementary information12.

## Data Availability

The datasets generated and/or analysed during the current study are available from the corresponding author on reasonable request.
