# Supplementary material for: Long-term imaging of the photosensitive, reef-building coral Acropora muricata using light-sheet illumination
Source: Sci Rep. 2020 Jun 25;10:10369. doi: 10.1038/s41598-020-67144-w (PMC7316744; doi:10.1038/s41598-020-67144-w)
Supplement: Supplementary file 1 — Supplementary information. [file 41598_2020_67144_MOESM1_ESM.pdf]

SUPPLEMENTARY TABLE 1

List of all Supplementary Videos

**Title**	**Corresponding figure**	**Description** (Note: Scale bars are denoted in the corresponding figure, unless otherwise indicated)
Supplementary Video S1	Fig. 2 A	L-SPI scanning process of a large part of the growing edge of a colony, containing multiple polyps. The image at the end is a maximum intensity projection of the scanned image stack.
Supplementary Video S2	Fig. 2 C	Time-lapse recording of small polyp over 4.7 h
Supplementary Video S3	Fig. 3 A, top row	Polyp emergence in low irradiance conditions (18.4 mW/cm^2) over 3h
Supplementary Video S4	Fig. 3 A, graph	Polyp emergence in brightfield conditions (6.2 mW/cm^2) over 3h (scale bar = 200 µm)
Supplementary Video S5	Fig. 3 A, bottom row	Polyp emergence in high irradiance conditions (82.6 mW/cm^2)
Supplementary Video S6	Fig. 3 B	Polyp switching from low to high irradiance. The polyp emerges at low irradiance (9.2 mW/cm^2) and retracts at high irradiance (59.7 mW/cm^2)
Supplementary Video S7	Fig. 4 A, B	Polyp, coral tissue and zooxanthellae in low light (9.2 mW/cm^2) over 6 h
Supplementary Video S8	Fig. 4 C	Close-up of zooxanthellae from Supplementary Video S7
Supplementary Video S9	Fig. 4 C	Tracking of the movement of zooxanthellae from Supplementary Video S7
Supplementary Video S10	Fig. 5	Tissue rupture at high irradiance (47.8 mW/cm^2) after 9 h
Supplementary Video S11	Fig. 6 A	Retracted polyps at the tip of a coral branch, imaged with CLSM
Supplementary Video S12	Fig. 6 C	Polyp imaged with WFM over 6 h

SUPPLEMENTARY TABLE 2

Lateral resolutions and light-sheet dimensions

a) Lateral resolutions using different detection objectives					
Magnifi-cation, type	*Numerical aperture*	*Theoretical Rayleigh resolution limit (µm, at 510 nm)*	*Measured in thick samples (µm, SD)*	*Manu-facturer*	*Objective model*
**4x, air**	0.1	3.1	4.1 ± 0.8	Olympus	PLAN 4X
**10x, water immersion**	0.3	1.0	1.4 ± 0.2	Olympus	UMPLFLN 10XW
**20x, water immersion**	0.5	0.6	0.7 ± 0.1	Olympus	UMPLFLN 20XW
**40x, water immersion**	0.8	0.4	0.6 ± 0.1	Olympus	LUMPLFLN 40XW
b) Light-sheet dimensions using different cylindrical lenses					
Focal length (mm)	*Measured waist (FWHM) (µm, SD)*	*confocal parameter (mm)*	*Maximum width (mm)*	*Manufacturer*	*Application*
**50**	20.7 ± 0.8	1.6	20	Thorlabs	For large FOVs
**30**	11.7 ± 0.8	0.6	20	Thorlabs	For single polyps

SUPPLEMENTARY TABLE 3
Irradiance conditions.

Technique	Detection objective magni-fication	Irradiance [mW/cm^2]	Photon flux [µmol m^{-2} s^{-1}]	Dura-tion [h, SEM]	Mode	Physiological effect
L-SPI (non-invasive, long-term)	0.5x – 40x	9.2	375	8.3 ± 0.8	Continuous image acquisition	Rapid polyp expansion; no long-term effects measured; slight photobleaching of coral tissue
L-SPI (non-invasive, short-term)	0.5x – 40x	18.4	750	3	Continuous image acquisition	Rapid polyp expansion; no long-term effects measured; slight photobleaching of coral tissue
L-SPI (invasive range)	0.5x – 40x	47.8 to 82.6	1948-3372	8.7h ± 0.3	Continuous image acquisition	Slow polyp expansion or retraction; pronounced photobleaching; tissue rupture, leading to loss of entire fragment
WFM	4x	1.0	253	6	5 min intervals	Pronounced photobleaching of coral tissue despite highly reduced spatiotemporal parameters
CLSM	10x	34’414	$1.4 * 10^6$	3	1 h intervals	Retraction of polyps and coral tissue, leading to tissue rupture and loss of entire fragment

SUPPLEMENTARY TABLE 4
Replicates and powers.

Figure(s)<br>Suppl. Figure(s)<br>Suppl. Video(s)	Experiment	Biological replicates (colonies)	Power [µW, SD]	Technique
Fig. 3 A (top row)<br>Suppl. Fig. S1<br>Suppl. Video S3	Polyp emergence at low irradiance	7 polyps in 3 replicates	19.7 ± 1.8	L-SPI, non-invasive, short-term, continuous
Fig. 3 A (graph)<br>Suppl. Video S4	Polyp emergence in low white light	7 polyps in 3 replicates	6.2 ± 2.3	Brightfield microscopy, non-invasive, continuous
Fig. 3 A (bottom row)<br>Suppl. Fig. S1<br>Suppl. Video S5	Polyp emergence at high irradiance	7 polyps in 3 replicates	89.8 ± 2.3	L-SPI, invasive, short-term, continuous
Fig. 3 B<br>Suppl. Video S6	Polyp dynamics from low to high irradiance	3 polyps in 3 replicates	10.2 ± 0.9<br>to<br>52.0 ± 4.2	L-SPI, invasive, short-term, continuous
Suppl. Fig. S2	Polyp dynamics from high to low irradiance	6 polyps in 3 replicates	52.0 ± 4.2<br>to<br>10.2 ± 0.9	L-SPI, invasive, short-term, continuous
Fig. 4<br>Suppl. Videos S7, S8, S9	Low irradiance imaging	6 replicates	10.2 ± 0.9	L-SPI, non-invasive, long-term, continuous
Fig. 4<br>Suppl. Fig. S3<br>Suppl. Video S7	Polyp dynamics at low irradiance imaging	3 replicates	10.2 ± 0.9	L-SPI, non-invasive, long-term, continuous
Suppl. Fig. S4	Controls for growth measurements	6 replicates	10.2 ± 0.9	Brightfield microscopy, non-invasive, continuous
Figs. 5, 6<br>Suppl. Fig. S4 (graph)<br>Suppl. Video S10	Tissue rupture at high irradiance	3 replicates	52.0 ± 4.2	L-SPI, invasive, long-term, continuous
Fig. 7 A<br>Suppl. Video S11	Polyp retraction at high irradiance (CLSM)	3 replicates	1.1 ± 0.5	CLSM, invasive, single timepoint
Fig. 7 B	Tissue contraction at high irradiance imaging (CLSM)	3 replicates	1.1 ± 0.5	CLSM, invasive, short-term, intervals (1h)
Fig. 7 C<br>Suppl. Video S7	Photobleaching (L-SPI)	3 replicates	10.2 ± 0.9	L-SPI, non-invasive, long-term, continuous
Fig. 7 C<br>Suppl. Video S12	Photobleaching (sparse WFM)	3 replicates	168 ± 3	Sparse WFM, non-invasive, Intervals (5 min)

SUPPLEMENTARY TABLE 5

Components required for building an L-SPI

Components list				
Optomechanical components:	**Product code**	**Quan-tity**	**Provider**	**Comment**
Postholders	UPH1.5	8	Thorlabs	
Posts	TRP1.5-P5	2	Thorlabs	
Kinematic mirror mount	POLARIS-K1	2	Thorlabs	placed before cylindrical lenses
1" Full Gimbal Mount	GMB1	1	Thorlabs	placed after beam expander and before rotating mirror for alignment control
Broadband Dielectric Mirror	BB1-E02	3	Thorlabs	
Lens mount for 1/2" optics	LMR05	2	Thorlabs	beam expander
Achromatic doublet f=25mm	AC127-025-A-ML	1	Thorlabs	beam expander
Achromatic doublet f=50mm	AC127-050-A-ML	1	Thorlabs	beam expander
Adjustable Mechanical Slit, Metric	VA100/M	1	Thorlabs	place after rotating mirror for varying width of light sheet
Kinematic Rectangular Optic Mount, Right Handed, Adjustable Height	KM100C	1	Thorlabs	
Kinematic Rectangular Optic Mount, Left Handed, Adjustable Height	KM100CL	1	Thorlabs	
Small Adjustable Clamping Arm, 6-32 Threaded Post	PM3	2	Thorlabs	
N-BK7 Plano-Convex Cylindrical Lens, f = 50.00 mm, H = 20.00 mm, L = 22.0 mm, Antireflection Coating: 350-700 nm	LJ1821L1-A	2	Thorlabs	For light-sheet dimensions, see Supplementary Table 1
N-BK7 Plano-Convex Cylindrical Lens, f = 30.00 mm, H = 20.00 mm, L = 22.0 mm, Antireflection Coating: 350-700 nm	LJ1212L1-A	2	Thorlabs	The shorter focal length of these cylindrical lenses requires that the kinematic mounts (KM100C and KM100L) are moved closer to the sample
Pedestal Pillar Post	RS1.5P	6	Thorlabs	4 for mounting heat sink and laser, 2 for z-stage
Small clamping fork	CF125	6	Thorlabs	4 for mounting heat sink and laser, 2 for z-stage
Ø1.5" Mounting Post Bracket	C1505	1	Thorlabs	for mounting microscope pillar (base removed)
50:50 Non-Polarizing Beamsplitter Cube, 400 - 700 nm, 5 mm	BS007	1	Thorlabs	
Right-Angle Prism Dielectric Mirror, 400 - 750 nm, L = 10.0 mm	MRA10-E02	1	Thorlabs	
Ø1", SM1-Mounted N-BK7 Ground Glass Diffuser, 600 Grit	DG10-600-MD	1	Thorlabs	for brightfield imaging
Right-Angle Prism Dielectric Mirror, 400 - 750 nm, L = 25.0 mm	MRA25-E02	1	Thorlabs	for brightfield imaging; position underneath hole of L-shaped bracket fitted to z-stage
Z-stage:				
Single-Axis, 0.98" Travel, Motorized Translation Stage	PT1-Z8	1	Thorlabs	
PT-Series Angle Bracket	PT102/M	1	Thorlabs	
T-Cube DC Servo Motor Controller (Power Supply Not Included)	TDC001	1	Thorlabs	
15 V Power Supply Unit for a Single T-Cube	TPS001	1	Thorlabs	
Motor Extension Cable, 2.5 m, DB15 Male to DB15 Female	PAA632	1	Thorlabs	
Laser:				
OBIS 488 nm LS 100 mW	OBIS 488 LS	1	Coherent, Inc.	
Rotating mirror:				
Panasonic AN8248NSB	n/a	1	Panasonic Corp.	See weblink below
375W Linear DC Variable Voltage Bench Power Supply	RP10L	1	Maplin	
Accessories:				
8-32 Cap Screw and Hardware Kit	HW-KIT1	1	Thorlabs	

1/4"-20 Cap Screw and Hardware Kit	HW-KIT2	1	Thorlabs	
Laser Safety Glasses	LG3	1	Thorlabs	
USB Temperature and Humidity Data Logger, -50 °C to 150 °C	TSP01	1	Thorlabs	
Additional External Temperature Probe, -15 °C to 200 °C	TSP-TH	1	Thorlabs	
Plain Glass Slides 76 mm x 39 mm x 1.0-1.2 mm (Pack of 100)	AGL4222A	1	Agar Scientific, UK	for observation vessel
Large Glass Slides 102 mm x 83 mm (Pack of 36)	AGL4380-2	1	Agar Scientific, UK	for observation vessel
AquaMate Silicone Sealant, fungicide and solvent-free	AQUATR	1	Everbuild, UK	for observation vessel
Monument Tools Fluorescein Drain Dye	31595	1	Screwfix	for lightsheet measurements

Rotating mirror from laser printer scanner assembly, available at:
https://www.itcsales.co.uk/cgi-bin/sh000002.pl?WD=scanner&PN=HP-LaserJet-1300-1150-3380-Scanner-Assembly-RM1-0524-7650%2ehtml#SID=473

Online instructions for controlling OBIS laser using Arduino and µManager:
https://actin.cn/2014/12/Arduino-UNO-control-OBIS-via-digital-trigger
https://micro-manager.org/wiki/Control_laser_shutters_with_Arduino

SUPPLEMENTARY FIGURES

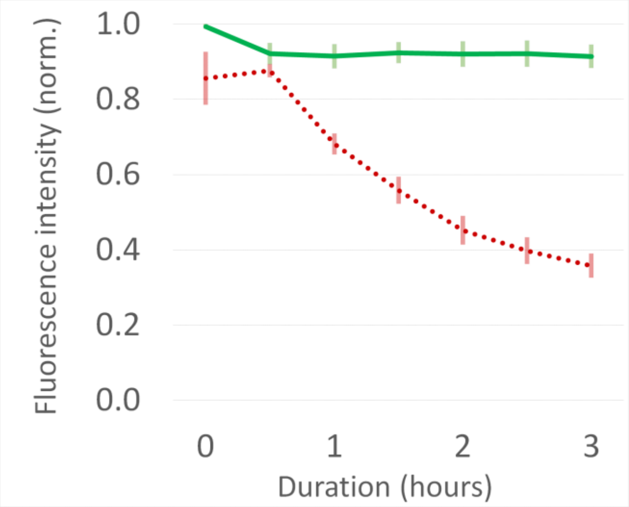

Supplementary Fig. S1 Photobleaching in *Acropora muricata* at low irradiance (green continuous line) and high irradiance (red dotted line).
Photobleaching was assessed by measuring fluorescence intensity over time. Since the polyps are highly dynamic, the measurements were restricted to the coenosarc (i.e. the coral tissue between polyps). In low light illumination (20 µW power, 18.4 mW/cm^2 irradiance) fluorescence intensity had decreased little, down to 92% ± 5% after 1.5 hours. After three hours, this had only decreased by another percent, down to 91% ± 3%. By contrast, fluorescence intensity in high light illumination (90 µW power, 82.6 mW/cm^2 irradiance) had decreased to almost half of its original value after 1.5 hours (56% ± 4%) and further down to 36% ± 3%. A total of three replicates were measured for each condition. Error bars indicate SEM.

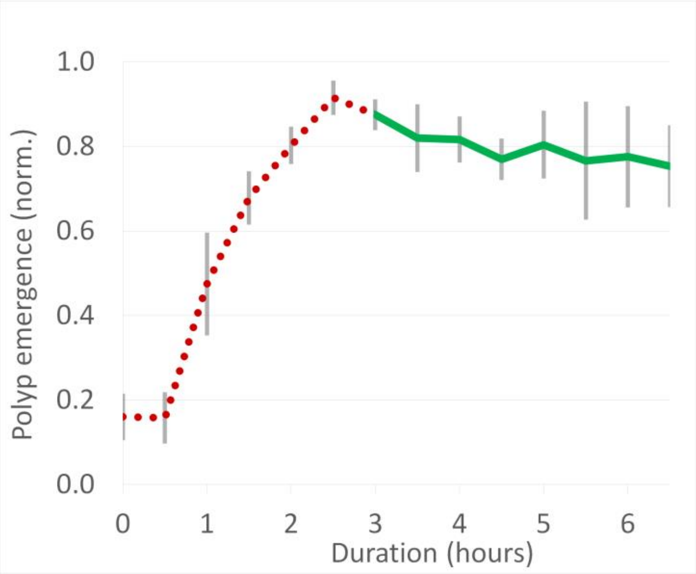

Supplementary Fig. S2 Polyp dynamics upon switching from high irradiance (red dotted line) to low irradiance (green solid line) after three hours. Polyps expanded slowly in high irradiance (59.7 mW/cm^2). Upon switching to low irradiance (9.2 mW/cm^2) after three hours, no polyp contraction occurred. A total of six polyps in three replicates were measured for each condition. Error bars indicate SEM.

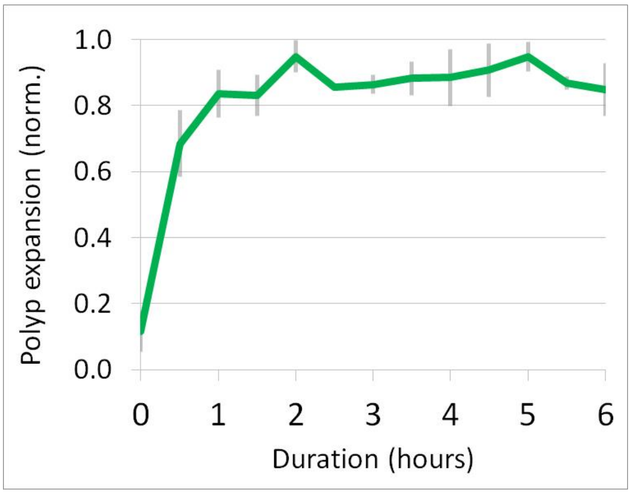

Supplementary Fig. S3 Polyp expansion remains unchanged over 6.5 hours continued image acquisition at low irradiance (9.2 mW/cm^2). The measurements here complement Figs. 3 and 4. A total of three polyps in three replicates were measured. Error bars indicate SEM.

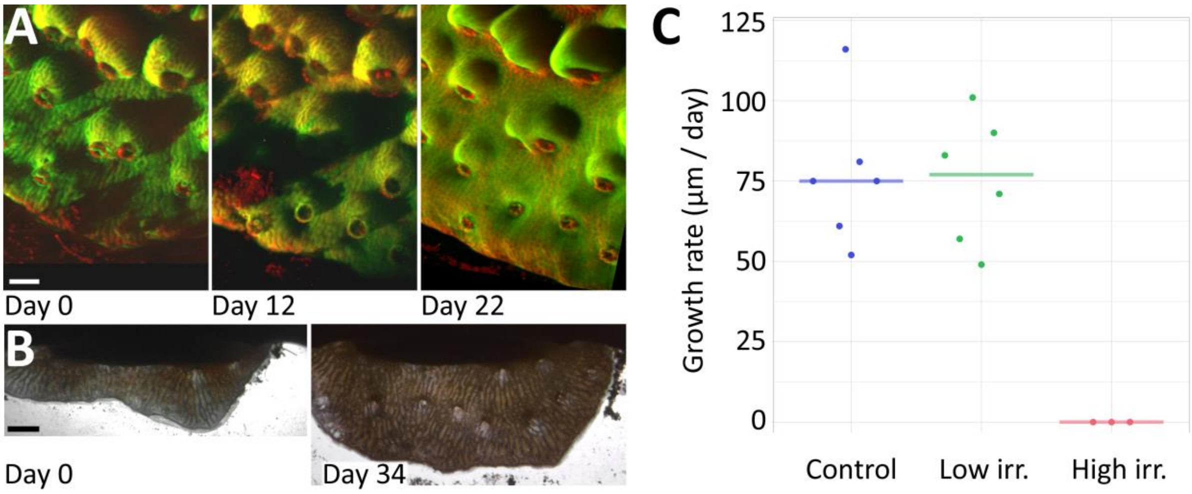

Supplementary Fig. S4 Growth rates of samples imaged in different conditions. A) Repeated imaging of the growing edge of a coral fragment imaged at low irradiance (9.2 mW/cm^2) using the L-SPI. Samples (n = 6) were subjected to 8.3 ± 0.8 hours of continuous image acquisition at least twice, receiving a minimum of 184 ± 20 mJ of exposure per imaging session. Scale bar = 1 mm. B) Control sample imaged using brightfield illumination. Scale bar = 1 mm. C) Graph showing brightfield-imaged controls ('control', blue) and samples imaged using light-sheet fluorescence microscopy at low irradiance ('Low irr.', green) and high irradiance ('High irr.', red, 59.7 mW/cm^2). Low irradiance light-sheet samples and controls were measured over 20 ± 1.5 days. High irradiance samples perished within two days after imaging. Dots represent individual samples, lines represent the median value.
